# A tonic electrocutaneous stimulation paradigm for graded sensory C-fiber engagement in healthy subjects

**DOI:** 10.3389/fpain.2026.1779451

**Published:** 2026-03-12

**Authors:** Youngsun Kong, Andrew G. Peitzsch, Riley Q. McNaboe, I-Ping Chen, Ki H. Chon

**Affiliations:** 1 Department of Biomedical Engineering, University of Connecticut, Storrs, CT, United States; 2 Department of Endodontics, University of Connecticut Health, Farmington, CT, United States

**Keywords:** C-fiber nociception, electrocutaneous stimulation, electrodermal activity, skin conductance, visual analog scale, temporal summation of pain, pain intensity, sympathetic nervous system

## Abstract

**Introduction:**

Long-term pain models provide valuable proxies for studying physiological changes associated with chronic pain, which is commonly associated with sustained C-fiber-mediated nociceptive input and central sensitization, and are particularly useful in therapeutic and drug development research. We investigated tonic electrocutaneous stimulation (ECS), a method that enables precise temporal control and adjustable intensity within safe limits, using a 5 Hz sine-wave waveform intended to bias sensory responses toward C-fibers-mediated activity.

**Methods:**

Twenty-one participants underwent three levels of personalized, 60-second ECS delivered at parameters intended to bias sensory responses toward C-fiber-mediated activity, three levels of short-term ECS using parameters commonly associated with Aδ-fiber-mediated responses, and mild long-term ECS using parameters commonly associated with Aβ-fiber-mediated activity as a non-painful control, skin conductance response (SCR) and level (SCL), continuous self-reported visual analog scale (VAS) pain ratings were recorded simultaneously.

**Results:**

The low-, medium-, and high-intensity ECS conditions produced statistically distinct self-reported pain scores and differentiable sympathetic nervous system (SNS) activation patterns in SCR and SCL. Compared to the non-painful control, low-intensity C-fiber-biased ECS did not differ in pain perception or SNS activity, whereas medium and high intensities elicited significantly greater responses. Repeated-measures correlation analysis revealed very strong associations between VAS scores and stimulus intensity across the entire stimulation period (*r ≥* 0.90, *p*<.001). Both SCR and SCL exhibited fair correlation coefficients throughout stimulation (*r* = 0.35-0.58, *p*<.05), while SCL correlations declined to poor, non-significant levels after first 30 seconds (*r* = -0.05-0.22, *p* = *n.s.*).

**Conclusion:**

Our proposed model provides a controllable and reproducible approach for inducing long-term, intensity-dependent pain in human subjects, offering a physiologically relevant experimental paradigm with high temporal precision.

## 1 Introduction

To better understand and develop tools for addressing complexity of pain (1), various pain-inducing modalities have been employed to simulate pain in human subjects. These experimental tools have advanced our understanding of pain mechanisms and supported the development of models and algorithms for pain quantification and management applications, including drug development, therapy evaluation, and psychology assessment (2–4). In particular, long-term (tonic) pain models can capture key features relevant to chronic pain mechanisms, including C-fiber-mediated signaling and central sensitization (2), providing valuable proxies of studying physiological changes associated with chronic pain–especially relevant for therapeutic and drug development research.

Thermal-based models are useful but have notable limitations. The cold pressor test involves immersing a hand in cold water for several minute and engage both myelinated Aδ- and unmyelinated C-fiber-medicated pain pathways (5, 6). However, the cold pressor test has shown inconsistent results in analgesic studies, and its gradual onset make it unsuitable for repeated testing within a 1–2 h session (2). Contact heat pain can be experimentally induced to bias responses toward C-fiber-mediated sensitization using paradigms such as dual heat pulses or temporal summation (7, 8). Nevertheless, the maximum safe temperature (50°C) restricts the induction of higher pain intensities and reduce data reliability, particularly when the stimulation area is small.

Mechanical stimulation can elicit both Aδ- and C-fiber-mediated pain (9), but prolonged application is limited by risks of bruising and ischemia (2). Chemical stimulation (e.g., capsaicin, mustard oil, and hypertonic saline) and endogenous stimulation (e.g., ischemic stimulation or exercise-induced pain) can also be effective for specific purposes; however, they are characterized by variable onset times, limited reproducibility upon repetition, and difficulty in calibrating pain intensity across multiple levels (2).

Electrical stimulation of the skin, electrocutaneous stimulation (ECS), is another approach commonly used to induce pain, offering high temporal precision, well-defined safety limits, and the ability to elicit individualized calibrated intensity levels (2). ECS can be delivered using different electrical waveforms, including sine-wave alternating current (AC) and brief-pulse, unidirectional direct current (DC) stimulation. Brief-pulse DC can induce rapid depolarization of peripheral nerve membranes with a sharp onset; however, prolonged unidirectional current may lead to depolarization block (10) and raise safety concerns (2). In contrast, AC-based ECS has been widely employed in tonic stimulation paradigms, where its periodic waveform provides repeated depolarizing drive over time, thereby facilitating sustained neural activation and temporal summation (11–13).

AC-based ECS can also provide preferential engagement of nerve fiber activity. In general, electrical stimulation bypasses receptors and directly activates nerve fibers (2, 14), leading to non-specific nociceptors activation. However, sensory nerve fibers—including Aβ, Aδ, and C fibers—differ in diameter, degree of myelination, and conduction velocity, leading to differential responses to electrical stimulation. Leveraging these biophysical differences, several studies have demonstrated that electrical stimulation using specific sinusoidal frequencies can preferentially engage distinct nerve fiber populations. In particular, sine-wave stimulation at 5 Hz has been shown to preferentially evoke C-fiber-mediated responses (13, 15–17).

Thus, ECS represents a promising pain model, as it allows flexible control of parameters such as subject-specific intensity, duration, and safety thresholds, while maintaining precise temporal resolution. This mechanism has been widely employed in clinical studies to characterize sensory dysfunction in patients by probing fiber-type-associated abnormalities through current perception threshold testing (18–23). A limited number of studies have examined varying stimulus intensities using continuous ECS for up to 24 s (12, 13). However, such relatively short stimulation durations may be insufficient to investigate sustained tonic activation of specific nerve fiber populations. Jonas et al. demonstrated sinusoidal ECS at 4 Hz for 60 s in human subjects; however, stimulation was delivered at a fixed intensity level, which did not account for inter-subject variability, such as various pain tolerance and physiological dynamics (11). Their study reported significant decreases in perceived pain over time, consistent with habituation or adaptation effects. Such designs may therefore be limited in their ability to support sustained tonic pain modeling.

Based on these limitations, we hypothesized that a tonic ECS paradigm combining longer stimulation durations with subject-specific, graded intensities would better support sustained and controlled engagement of C-fiber-mediated sensory responses compared with conventional continuous stimulation. Accordingly, we propose a new paradigm of tonic ECS with varying stimulation intensities designed to bias sensory responses toward C-fiber-mediated activity and to evaluate their temporal dynamics in human subjects. This approach allows flexible intensity modulation to better characterize inter-subject variability, while enabling sustained stimulation that may approximate aspects of tonic pain relevant to certain chronic pain conditions. By supporting scalable data collection across large cohorts, this paradigm is particularly relevant for chronic pain research and translational studies, including drug and therapy development, and for providing new insights into fiber-specific physiological responses. Additionally, it can help capture key features relevant to chronic pain mechanisms, including sustained nociceptive drive biased toward C-fiber–mediated activity, temporal summation, and inter-individual variability.

## 2 Methods

### 2.1 Participant recruitment

Twenty-one healthy participants (14 males and 7 females; age 24 ± 4.3) were recruited. Participants were eligible if they were 18 years of age or older. Exclusion criteria included chronic cardiovascular disease; use of any medications affecting pain perception, autonomic nervous system, and sweat gland activity; pregnancy; Raynaud's syndrome; adhesive allergy; presence of a pacemaker or defibrillator; recent head trauma; and insensitivity to physical pain. Consent forms were obtained from all participants prior to each experiment (Protocol H23-0183).

Sample size (*N* = 21) was selected based on precedent in prior human ECS studies employing comparable stimulation paradigms and durations, which typically included cohorts of 11–16 participants. For example, our prior short-duration ECS study using a multi-level stimulation scheme (*N* = 16) (24), a study by Jonas et al. employing 60-s sinusoidal ECS (*N* = 14) (11), and a study by Matsubara et al. using varying stimulation intensities for durations up to 24 s (*N* = 11) (12).

A sensitivity power analysis was also performed for the primary within-subject VAS contrast between the highest pain condition and the non-painful reference condition (*α* = 0.05, two-sided). With *N* = 21 participants, the study has 80% power to detect a moderate within-subject effect size (Cohen's $d_{z}\approx 0.64$). Secondary outcomes were reported with effect sizes and 95% confidence intervals.

### 2.2 Stimuli and materials

**Figure 1:**
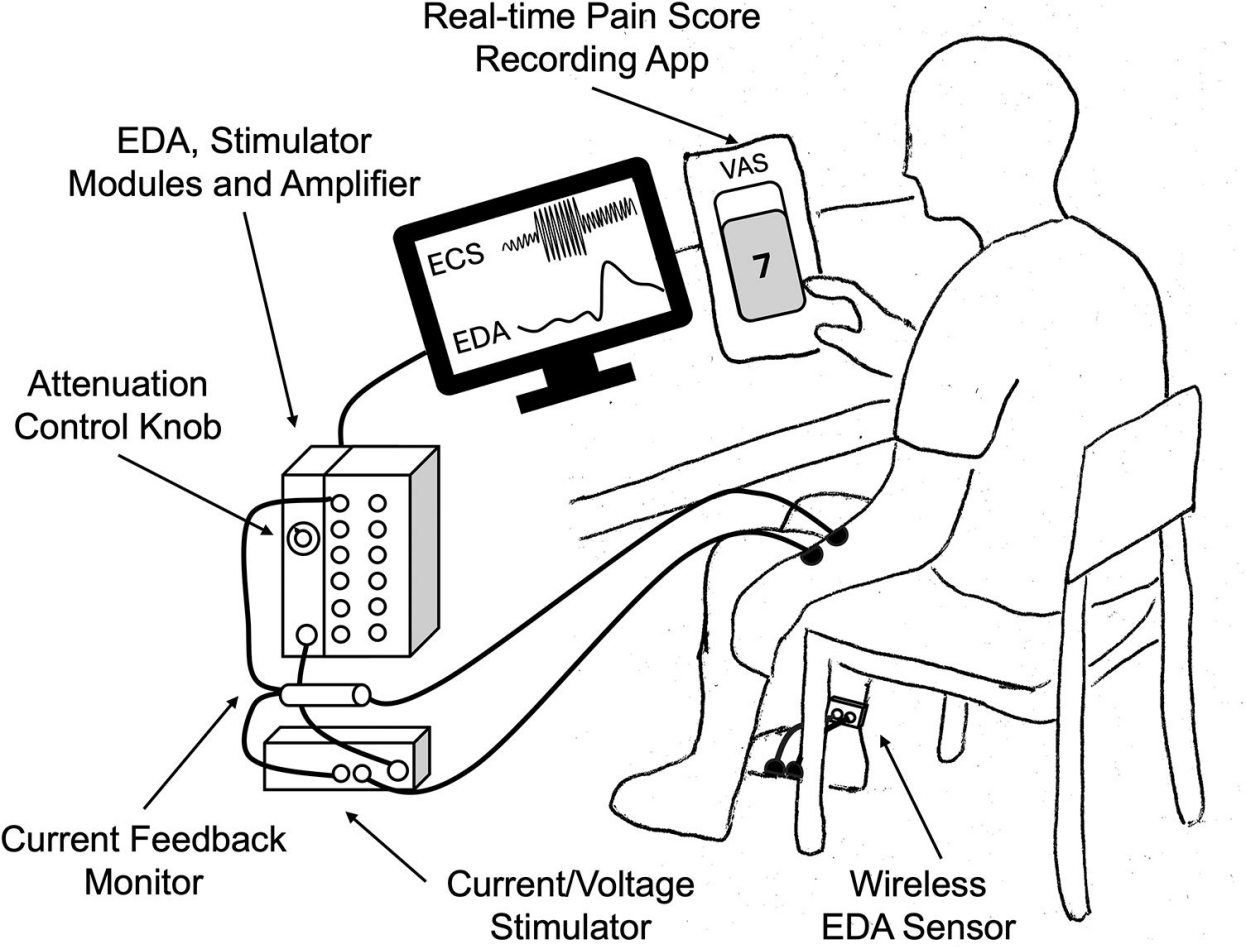
Experiment setup.

The experiment involved ECS with various personalized stimulation intensities and three waveform frequencies while simultaneously recording electrodermal activity (EDA) and self-report continuous pain scores through a custom smartphone application (Figure 1).

#### 2.2.1 Nerve fibers and electrocutaneous stimulation

Nerve fibers exhibit distinct responses to electrical stimulation depending on their diameter, degree of myelination, and conduction velocity, resulting in frequency-dependent activation patterns. Langille et al. investigated frequency-dependent biasing of sensory nerve fiber responses using sinusoidal stimulation (17). Large-diameter, heavily myelinated Aβ-fibers, which mediate fast, non-nociceptive sensory transmission, are preferentially activated at higher frequencies (2 kHz). Smaller, thinly myelinated Aδ-fibers are preferentially engaged at stimulation frequencies around 250 Hz, whereas unmyelinated C-fibers are most strongly engaged at low frequencies near 5 Hz. However, overlapping activation thresholds and sensitivities under low frequency stimulation can lead to concurrent involvement of Aδ- and Aβ-fibers, as observed in other pain models (17).

Although ECS does not enable exclusive activation of individual nerve fiber types, frequency- and intensity-dependent stimulation parameters can bias sensory responses toward specific fiber populations, as supported by prior studies (13, 15–17), including evidence from human studies using verbal pain descriptors, sensory threshold changes during cold pressor and tourniquet ischemia tests (13, 15, 25). Additionally, this mechanism has been employed to characterize sensory dysfunction and pain mechanisms by probing fiber-type-associated abnormalities using current perception threshold testing, including poststroke pain (23), diabetic neuropathy assessment (19), lower urinary tract (18), cervical myelopathy (22), restless legs syndrome/Willis–Ekbom disease (20), and lower extremity fractures (21).

In our study, ECS was delivered using BIOPAC STIMSOLA and STM100C programmable module (BIOPAC Systems, Inc., Goleta, CA). Two standard Ag/AgCl electrodes were placed on the volar surface of the non-dominant forearm for each participant, 2–3 cm apart. ECS conditions included sine-wave stimulation at 5 Hz, 250 Hz, and 2 kHz, commonly associated with C-fiber-, Aδ-fiber-, and Aβ-fiber-mediated sensory responses, respectively. These are referred to as the C-fiber, Aδ-fiber, and Aβ-fiber settings, respectively, in the rest of the paper.

For the C-fiber and Aδ-fiber settings, stimulation was applied at low, medium, and high intensity levels, whereas the Aβ-fiber setting included only low and medium levels. However, only the low-intensity Aβ-fiber condition was analyzed to serve as a non-painful control, given its association with predominantly non-nociceptive sensory responses. The Aδ-fiber setting served as a control condition to assess the contribution of Aδ-fiber-associated activity relative to the C-fiber setting. To induce varying intensities, the individual maximum current (I_max_) was determined separately for each setting. I_max_ corresponded to the current intensity producing a self-reported visual analog scale (VAS) score 7 out of 10 (Table 1). For the C-fiber and Aδ setting, low, medium, and high stimulation levels corresponded to 33%, 67%, and 100% of I_max_, respectively. The low Aβ-fiber stimulation was delivered using 33% of I_max_, because higher intensity settings (i.e., 67% and 100%) would likely involve co-activation of other fiber populations. To determine each I_max_, experimenters incrementally adjusted using the attenuation control knob on the STM100C unit until the participant reported the target VAS pain scores, with stimulation delivered in 10-second intervals.

**Table 1:** ECS settings. I_max_ indicates the individual maximum current for each setting.

Fiber setting	Waveform frequency	Duration	Self-report pain score	Level 1 (Low)	Level 2 (Medium)	Level 3 (High)	Remarks
C	5 Hz	60s	Continuous VAS	33% I_max_	67% I_max_	100% I_max_	
Aδ	250 Hz	10s	Single VAS after each stimulus	33% I_max_	67% I_max_	100% I_max_	Control: to assess the involvement of Aδ fibers during initial stimulation
Aβ	2 kHz	60s	Continuous VAS	33% I_max_	Not used	Not collected	Control: non-nociceptive tonic stimulation

For the C-fiber and Aβ-fiber settings, stimulation was applied for 60 s, whereas the Aδ-fiber setting was applied for 10 s at each intensity level. Aδ-fiber stimulation was delivered for 10 s in 0.5-s bursts, as longer stimulation durations may reduce perceptual salience due to habituation. For each waveform frequency, three randomized trials were conducted per intensity level, with inter-stimulus intervals randomly varied between 20 and 45 s.

The experimenters ensured participant safety by continuously monitoring the actual current delivered using a CBLCFMA current feedback monitor cable and INISOA signal isolation adapters (BIOPAC Systems, Inc, Goleta, CA, USA). The order of ECS settings was randomized for each participant, and a 10-minute rest period was provided between settings.

#### 2.2.2 Self-reported pain scores

An 11-point visual analog scale (VAS; zero being no pain and ten being the most severe pain) was used to individualize pain intensity for each ECS setting and to record pain responses to each stimulation event (26). Because the Aδ-fiber setting involved short-term stimulation (10 s) whereas the other settings involved long-term stimulation (60 s), collecting a single VAS score was not optimal. Therefore, we developed a custom smartphone application that allowed participants to continuously record their perceived pain in real time at 10 Hz (i.e., the application continuously read the value of a pain score slider at 10 Hz). Participants adjusted a vertical on-screen slider to indicate pain intensity, moving upward to represent higher pain levels and downward to represent lower levels, using their dominant hands. The screen displayed a near full-height slider bar with a number scale (0–10) corresponding to the VAS score. Details of the application validation are provided in the Supplementary Material.

#### 2.2.3 Electrodermal activity

EDA has been widely used to assess SNS activity during pain across various experimental modalities (27). EDA can provide valuable insight into temporal autonomic dynamics, as its signal can be decomposed into phasic (fast) and tonic (slow) components, representing skin conductance responses (SCRs) and skin conductance level (SCL) changes over time–corresponding to discrete bursts of sympathetic activity and overall sympathetic tone, respectively (28). In particular, the amplitude of the phasic component in response to pain stimuli and the gradual amplitude change of the tonic component have shown moderate correlations with stimulus intensity (29, 30).

To minimize ECS-noise caused by involuntary muscle contractions, EDA was recorded from the lateral arch area of the dominant hand-side foot, with electrodes placed ∼1 inch apart. EDA signals were acquired at a 2 kHz sampling rate using a BIOPAC BN-PPGED module (BIOPAC Systems, Inc, Goleta, CA, USA). Note that this location has previously been shown to yield EDA signals highly correlated with those recorded from the hand (31).

Raw EDA signals were then resampled to 4 Hz and lowpass filtered at a 1 Hz cutoff frequency to remove high-frequency noise. The cvxEDA approach was used to decompose the signals into phasic and tonic components, enabling extraction of SCRs and SCL, respectively (48). This method was selected because it has demonstrated superior classification performance for differentiating pain intensities compared with other decomposition techniques (24). To account for baseline variability, the SCL signals were normalized by setting the onset of each stimulus to zero within each trial segment, yielding the normalized SCL (nSCL).

### 2.3 Design and procedure

**Figure 2:**
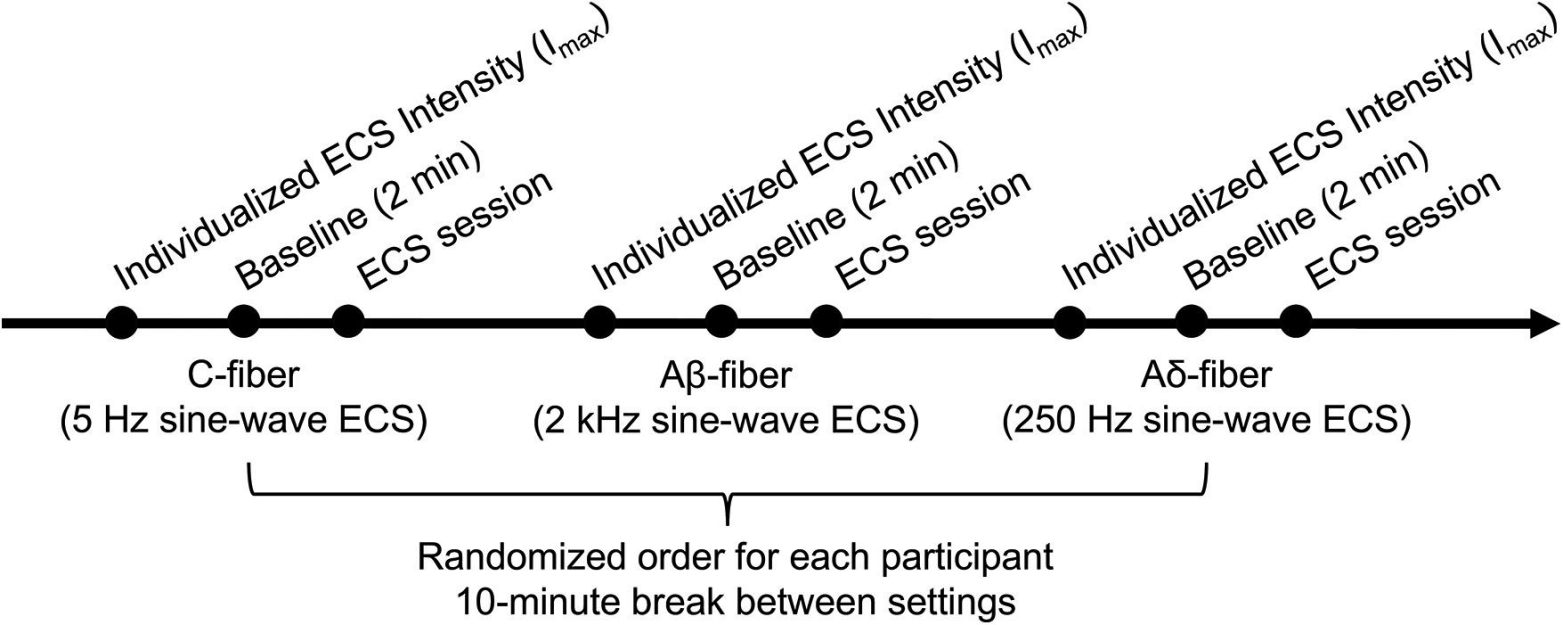
An example of experiment sequence.

All participants were instructed to abstain from any stimulating or caffeine-containing drink or food for at least 24 h before their experiment session. Upon arrival, participants completed a screening questionnaire to ensure eligibility and safety, minimizing confounding factors such as medications that could influence sweat gland activity. Written informed consent was then obtained. Participants were subsequently trained to use the smartphone application for continuously recording VAS pain scores. Electrodes were then placed for ECS and EDA recording, after cleaning the skin with 70% alcohol. Next, individualized stimulation intensity was determined for each participant, followed by a 2-minute baseline period to allow physiological function to return to resting state. An example of experiment procedure is shown in Figure 2.

Participants were informed that they could terminate the experiment at any time. If a participant requested to stop, the experimenters immediately turned the stimulation knob to zero. The actual current delivered was continuously monitored by the experimenters. All experiments were conducted in a 10 × 10-meter lab in the Engineering and Science building on the Storrs campus of the University of Connecticut. Participants were recruited between November 29 and December 18, 2023, and completed a single study visit with no follow-up. This research complied with tenets of the Declaration of Helsinki and was approved by the Institutional Review Board at the University of Connecticut (protocol H23-0183).

### 2.4 Segmentation and statistics

EDA signals and continuous VAS scores were segmented into 10 s with 5-second overlaps, resulting in one segment for Aδ-fiber setting and 11 segments for the other settings. Each segment was then averaged. For the Aδ-fiber setting, post-reported single VAS scores were used. Because the last segment was often truncated by a couple of seconds (due to unknown device issue), the 11th segment was discarded for the Aβ- and C-fiber settings for consistency.

Significance testing was performed to evaluate the main effects of Time (sessions), Intensity, and their interaction within the C-fiber setting, in comparison with the Aδ- and Aβ-fiber control settings. Three intensities of Aδ-fiber were set to baseline segment (Time factor), while 10 segments of Aβ-fiber were set to baseline intensity (Intensity factor). A linear mixed-effects model (LMER) was fitted to account for missing data, and Type III ANOVA with Kenward-Roger approximation was conducted. The model included a random intercept for participants, as adding random slopes for time did not improve model fit and resulted in singularity:

VAS,SCR,orSCL∼Time×Intensity+(1|Participant)

Post hoc pairwise comparisons were performed using the emmeans package with Dunnett correction for multiple testing (32), as each intensity level and time segment was compared against its respective reference condition.

For the continuous VAS data, we also computed slopes representing the rate of VAS change (ΔVAS/s) for each C-fiber intensity level to evaluate the effect of temporal summation (“wind-up”), a phenomenon in which repeated, identical nociceptive stimuli elicit a progressively increasing perception of pain (4, 33). Here, the Time factor was treated as a numeric variable. The corresponding LMER model accounted for both random intercepts and slopes across participants:

VAS∼continuousTime×Intensity+(1+continuousTime|Participant)

Finally, repeated-measures correlation coefficients between VAS and EDA features, as well as between VAS and stimulation intensity, were calculated for each time point under the C- and Aδ-fiber settings using the RMCORR package in R (34). The Aβ fiber setting was not included in this analysis. Interpretation of correlation coefficients were based on Chan et al. (35, 36). Statistical significance was defined as *p* < .05.

Because repeated measurements were obtained across time points within each participant and condition, observations were not independent. This within-subject non-independence was addressed using linear mixed-effects models with participant included as a random effect. Model assumptions were assessed using residual-vs.-fitted and normal Q–Q plots; diagnostic results are provided in the Supplementary Material.

## 3 Results

### 3.1 Participants

A total of 21 healthy participants were included in the study (14 males and 7 females, 24 ± 4.3 years old). Data from two participants were incomplete. One participant did not record continuous VAS ratings on the smartphone application during the C-fiber setting, and another withdrew early during the C-fiber Intensity 3 stimulation, providing only the first nine segments out of ten.

### 3.2 Self-reported pain scores

A two-way repeated-measures ANOVA with factors Time and Intensity showed a significant main effect of Intensity [*F*(3,811) = 1,402, *p* < .001], while the main effect of Time and the Time × Intensity interaction were not significant, suggesting that intensity effects were stable across timepoints.

Within each time point, VAS scores from intensity levels 2 (medium) and 3 (high) of C-fiber setting stimulation, exhibited significantly higher scores than control (Aβ-fiber, *p* < .001). In other words, C-fiber setting stimulation with intensity 1 (low) did not induce sufficient VAS that is differentiable from Aβ-fiber. Although the overall main effect of Time was not significant, our exploratory pairwise comparisons within intensity level 3 revealed that VAS scores recorded during all timepoints but first 5 s (S2–S10) were significantly higher than control (Aδ-fiber, *p* < .001). This suggests that temporal changes were present only at the highest intensity level, but the overall Time effect across all intensities, likely indicating the effect of temporal summation of pain for intensity level 3 (4, 33). This is also supported by our slope analysis. At stimulation level 3, the estimated slope of reported score over time was 0.016 units per second [95% CI (0.005, 0.027)], indicating a clear increasing trend. In contrast, levels 1 and 2 showed no meaningful temporal change, with slopes of 0.005 [95% CI (–0.016, 0.016)] and −0.005 [95% CI (–0.016, 0.006)], respectively, both confidence intervals including zero. Aβ-fiber control also exhibited no meaningful temporal change, with slopes of −0.008 [95% CI (−0.016, 0.006)]. This is summarized in Table 2 and Supplementary Figure S1.

**Table 2:** Statistical analysis and slope analysis on VAS.

Intensity	Segments with higher VAS than Aβ	Segments with higher VAS than Aδ	Slope (VAS/sec) [95 CI]
Aβ-fiber control	NA	NA	−0.008 [−0.016, 0.006]
C-fiber Level 1			−0.005 [−0.016, 0.006]
C-fiber Level 2	1–10 **		0.005 [−0.006, 0.016]
C-fiber Level 3	1–10 **	2–10 *	0.016 [0.005, 0.027]

* *p* < .05,

** *p* < .001.

**Figure 3:**
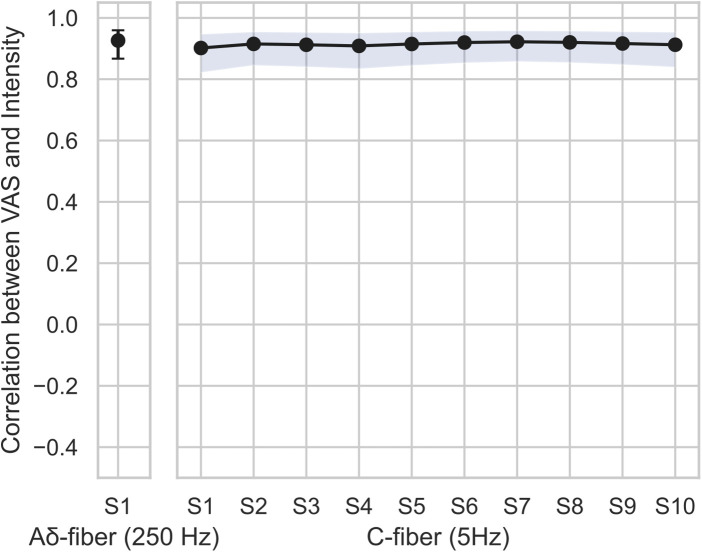
Repeated measures correlation coefficients between intensity and VAS for each time point. The error-bars and blue shades indicate 95% confidence intervals. All timepoints showed significant correlation (*p* < .001).

Our correlation analysis revealed a very strong effect size for the Aδ fiber setting (*r* = 0.93, *p* < .001). Within the C-fiber setting, all repeated-measures correlation coefficients were very strong (*r* ≥ 0.90, *p* < .001), as summarize in Figure 3. These results indicate that, across time, the personalized current intensities consistently elicited stable pain ratings for each participant.

### 3.3 Electrodermal activity analysis

For nSCL, a two-way repeated-measures ANOVA with factors Time and Intensity showed significant main effects of Intensity [*F*(3,839) = 25.8, *p* < .001], Time [*F*(10,839) = 3.6, *p* < .001], and their interaction [*F*(29,839) = 2.6, *p* < .001]. SCL changes (nSCL) over time showed were significantly higher during segments 2 and 3 (i.e., 10–25 s) compared with the Aδ-fiber control setting, but only at intensity level 3 (*p* < .05). Similarly, intensity level 3 also showed significantly elevated nSCL during segments 1–4 (i.e., 0–25 s) compared with the Aβ-fiber control (*p* < .05). These results provide additional evidence of temporal summation or sensitization effects (4, 33), with apparent saturation of SNS activation at later timepoints.

For SCR, a two-way repeated-measures ANOVA with factors Time and Intensity revealed a significant main effects of Intensity [*F*(3,811) = 44.7, *p* < .001] and Time (*F*(10,839) = 2.5, *p* = .005, while their interaction was not significant. SCR amplitudes were comparable between the Aδ- and C-fiber settings, whereas most segments at intensity level 3 (timepoints 2–4 and 6–9, around 5–50 s) showed significantly higher activity compared with the Aβ fiber control setting (*p* < .05). These results suggest that overall sweat gland activity was affected primarily during the first 25 s, whereas SCRs were significantly elevated throughout most of the stimulation period at the highest Intensity. These are summarized in Table 3; Supplementary Figure S2.

**Table 3:** Statistical analysis on EDA.

EDA component	C-fiber intensity	Segments with higher EDA than Aβ	Segments with higher EDA than Aδ
nSCL	1		
	2		
	3	1–4 *	2,3*
SCR	1		
	2		
	3	2–4*, 6–9 *	

* *p* < .05.

**Figure 4:**
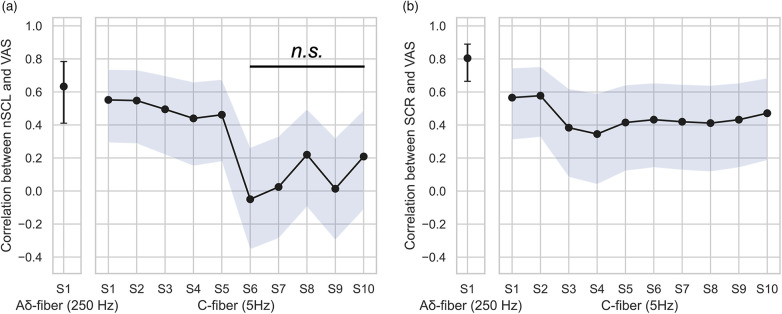
Repeated measures correlation between **(a)** nSCL and VAS, and **(b)** SCR and VAS at each time point. The error-bars and blue shades indicate 95% confidence intervals. All timepoints in panel **(a)**, and all time points in panel **(b)** except those between S6 and S10, showed significant correlations (*p* < .05).

These relationships were further examined through repeated-measure correlation analysis (Figure 4). For nSCL, a stronger association was observed in the Aδ fiber setting compared with all timepoints in the C-fiber setting (*r* = 0.63, *p* < .001). Within the C-fiber setting, correlation coefficients were fair during the first 30 s (*r* = 0.46–0.55, *p* < .05) but decreased to poor levels (*r* = −0.05–0.22, *p* = n.s.) thereafter. For SCR, correlation analysis also revealed a higher effect size in the Aδ-fiber setting compared with all timepoints of the C-fiber setting (*r* = 0.8, *p* < .001). In contrast, the C-fiber setting showed fair correlations across most time points (*r* = 0.35–0.58, *p* < .05).

## 4 Discussion

We proposed our new tonic ECS paradigm for graded engagement of C-Fiber-mediated sensory responses and evaluate their temporal dynamics. We applied sine-wave ECS at 5 Hz on the volar forearm at three individualized intensity levels (low, medium, and high) to bias neural responses toward C-fiber-mediated activitiy, and its effectiveness was evaluated using self-reported VAS scores and EDA signals. For comparison, sine-wave ECS at 250 Hz was applied to bias responses toward Aδ-fiber-mediated activity, and low-intensity ECS at 2 kHz was used to bias responses toward activate Aβ-fiber-mediated activity as a non-painful long-term reference condition.

We found that low-intensity stimulation of the C-fiber setting did not differ statistically from the Aβ-fiber setting (touching sensation) in terms of self-reported pain, whereas medium- and high- intensity stimulations elicited significantly higher self-reported VAS scores. Notably, high-intensity stimulation of the C-fiber setting produced significantly greater pain ratings than that of Aδ-fiber setting. These findings suggest that low-intensity stimulation of the C-fiber setting may not reach the pain threshold. Although the main effect of Time was not significant, high-intensity stimulation exhibited an increasing trend over time, reflecting a tendency toward temporal summation effects that was not observed in lower intensities or in the Aβ-fiber control setting. This weak evidence of temporal summation of pain may be because that classic “wind-up” phenomena typically emerge when repeated nociceptive stimuli are delivered at intervals of 3 s or less (37), whereas our study employed continuous sine-wave stimulation at 5 Hz (every 0.2 s). Although electrical stimulation studies have used frequencies of 0.3 Hz and higher to investigate temporal summation of pain (38), it is possible that the effects we observed are constrained by technical or methodological limitations inherent to electrical stimulation. For example. In the study by Enax-Krumova et al., participants exhibited an increasing trend in pain ratings during painful cutaneous electrical stimulation with short intervals (three monopolar square waves that last for 500 µs each with 5 ms interval in between), but the effect did not reach statistical significant (39). This should be further explored in future studies.

A similar pattern was observed in the EDA analyses. For both SCR and nSCL measures, low- and medium-intensity stimulations did not differ significantly from the reference Aβ-fiber setting. However, high-intensity stimulation produced statistically significant increases compared with the Aβ-fiber setting. In particular, nSCL showed significant increases during the first ∼30 s and subsequently declined, with correlation effect sizes decreasing from moderate to weak beyond 30 s. We have two assumptions. The first assumption is partial co-activation of Aδ fibers at higher stimulation intensities. Previous studies–primarily focused on short-term stimulation–that reported partial Aδ-fiber involvement during 5 Hz ECS (17, 40). A transient shift from Aδ- to C-fiber-mediated responses has also been described in pain models such as contact heat, cold pressor, pressure stimulations (2, 41, 42). The other assumption is sympathetic adaptation, a phenomenon frequently observed during sustained stress or pain exposure (43). For instance, a recent study reported that EDA initially increased and then gradually decreased during the cold pressor test (44). Future studies should further investigate these mechanisms by employing multimodal autonomic and neuroimaging measures.

ECS has been employed to evoke painful sensations and simulate pain responses for clinical model development. One study adopted a similar approach using short-term direct current electric pulse with the 10 *ms* width to induce four individualized pain levels (25%, 50%, 75%, 100% of each participant's maximum current based on VAS 7 out of 10) on the volar forearm, aiming to develop machine learning models for autonomic pain differentiation based on EDA signals (45). Their model demonstrated a strong correlation between VAS ratings and stimulus intensity (*r* = 0.94) and increased SNS activation as indicated by EDA analysis. Other investigators have also explored frequency-based ECS paradigms to target specific nerve fibers using multi-level stimulation approaches. Tsuji, Yamawaki, and their colleagues applied ECS at 5 Hz and 250 Hz on the volar forearm to bias responses toward C-and Aδ-fiber-mediated activity, respectively (12, 46). Their study design defined baseline stimulation as the current producing a numeric rating scale score of 3 out of 10 with three multipliers (0.5×, 1×, 1.5×) to modulate pain levels. They reported strong correlations between VAS ratings and stimulus intensity (R^2^ = 0.98 for C-fiber and 0.94 for Aδ-fiber) (12), along with increased SNS activity for both conditions as assessed via the arterial stiffness index. Notably, the average pain scores at high intensities in their study were slightly lower than those observed in ours, likely due to differences in the baseline current intensity calibration scheme.

### 4.1 Limitations and future directions

Our study was conducted in healthy young participants (undergraduate and graduate students), where our approach can be extended to a broader range of cohorts. Future studies should validate these models in diverse populations, as human physiological signals are inherently variable due to factors, such as perspiration rate, pain threshold, hydration status, and other interindividual differences. These considerations are essential for improving the generalizability and robustness of ECS-based pain and autonomic modeling. Additionally, the study sample was imbalanced with respect to biological sex. Exploratory analyses in the Supplementary Material revealed a modest sex-by-intensity interaction but no significant main effect of sex. Because the present study was not designed or powered to characterize sex-specific response patterns, these findings should be further investigated in larger, balanced cohorts.

In addition, we employed conventional ECS settings in this study, using a sine-wave signal and two electrodes placed approximately 1.5 cm apart on the forearm. However, alternative configurations may further improve accuracy and reproducibility. Imatz-Ojanguren and Keller systematically compared stimulation frequencies (5 Hz, 250 Hz, and 2 kHz), waveforms (sinusoidal, square, and 250 µs-pulsed), and electrode configurations (conventional vs. concentric) in human subjects when assessing current perception thresholds. They concluded that both waveform and frequency parameters can significantly influence pain perception (47). Such parameter variations should be carefully considered in future ECS studies to optimize stimulus specificity and reliability. Finally, there are still limited knowledge on the involvement of fibers in our settings. Most frequency-based ECS studies are based on current perception thresholds, which typically does not go to the high intensity settings. A direct and accurate method to verify fiber-specific activation would be microneurography; however, electrical stimulus inheritably corrupts microneurography recordings because the signals are highly susceptible to electrical noise. Future studies should further validate our paradigm using multimodality including neuroimaging.

Several additional methodological considerations should be noted. Continuous interaction with the smartphone slider may have introduced attentional effects; however, minimal EDA responses at low stimulation intensities suggest that any such influence was likely limited. We also did not observe evidence of habituation or adaptation, in contrast to prior work using fixed-intensity stimulation that reported significant decreases in reported-pain scores over time (11). In the present study, VAS ratings did not show a systematic decrease over time, and an increasing trend was observed at the highest stimulus intensity. Future studies with longer stimulation durations may further clarify potential adaptation effects. Finally, although EDA is well suited for tracking SNS activity with high temporal sensitivity—particularly in ECS-based paradigms where electrical noise can limit other signals—future studies may benefit from incorporating complementary autonomic measures, such as respiration, to provide a more comprehensive assessment of autonomic regulation.

## 5 Conclusion

We proposed and evaluated an experimental paradigm to optimize tonic ECS delivered at parameters intended to bias sensory responses toward C-fiber-associated activity. Our three-level stimulus paradigm–designed to induce personalized low, medium, and high levels of long-term pain–produced statistically distinct self-reported pain scores, as confirmed by correlation analysis, and differentiable SNS activation, as verified through EDA analysis. Comparisons with the Aβ-fiber long-term setting indicated that medium- and high-intensity stimulations elicited clear increase in both perceived pain and SNS activation, whereas mild stimulation did not, likely because it did not reach the pain threshold.

Overall, tonic ECS with 5 Hz sine-wave stimulation provides an effective approach for inducing long-term, intensity-dependent pain in human subjects, with the initial ∼30 s likely influenced by transient Aδ-fiber-associated responses. We anticipate that this paradigm can contribute to the development of more accurate and physiologically grounded pain models, enabling improved assessment of therapeutic and pharmacological interventions. By enabling controlled and reproducible induction of graded pain with high temporal precision, ECS-based frameworks such as ours may provide a useful experimental platform for investigating pain mechanisms and informing future translational studies.

## Data Availability

The raw data supporting the conclusions of this article will be made available by the authors, without undue reservation.
