# Supplementary material for: A tonic electrocutaneous stimulation paradigm for graded sensory C-fiber engagement in healthy subjects
Source: Front Pain Res (Lausanne). 2026 Mar 12;7:1779451. doi: 10.3389/fpain.2026.1779451 (PMC13017951; doi:10.3389/fpain.2026.1779451)
Supplement: Supplementary file 1 [file Datasheet1.pdf]

Supplementary Material

A Tonic Electrocutaneous Stimulation Paradigm for Graded Sensory C-Fiber Engagement in Healthy Subjects

1 Supplementary Figures

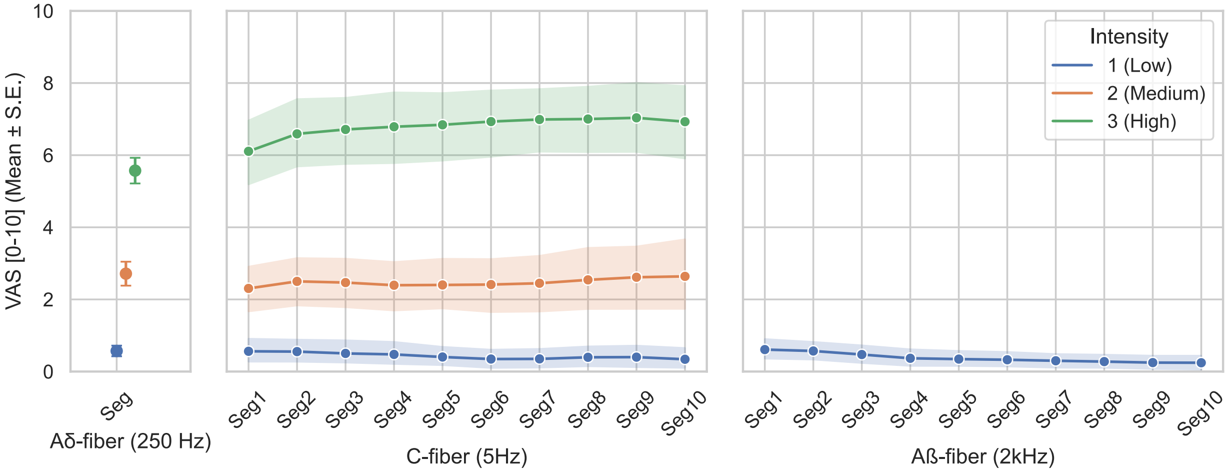

Supplementary Figure 1. Mean ± SEM of self-reported pain intensity (VAS) across time segments for each ECS intensity. Data points represent group means; error bars indicate between-subject variability. VAS: visual analog scale (0–10).

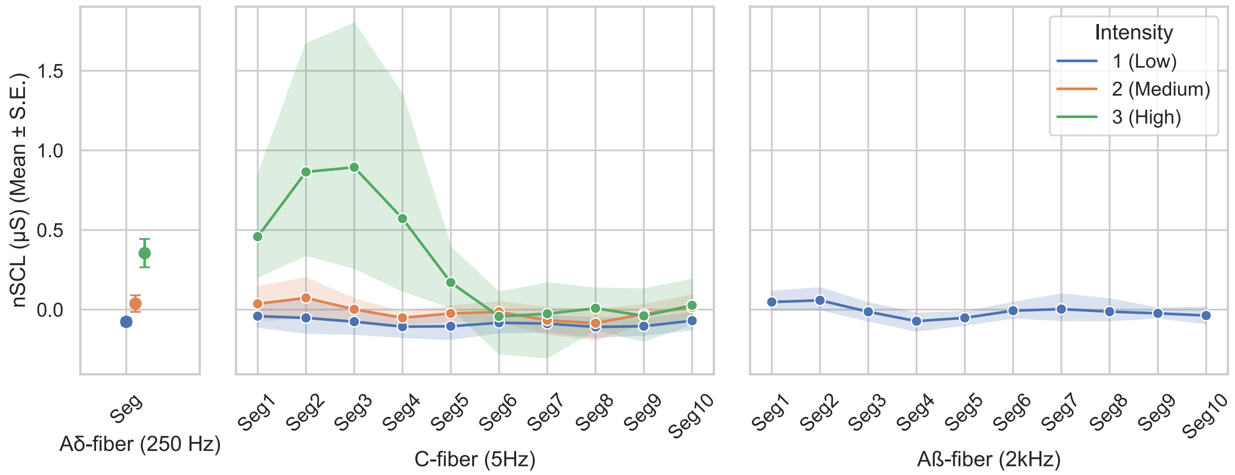

(a)

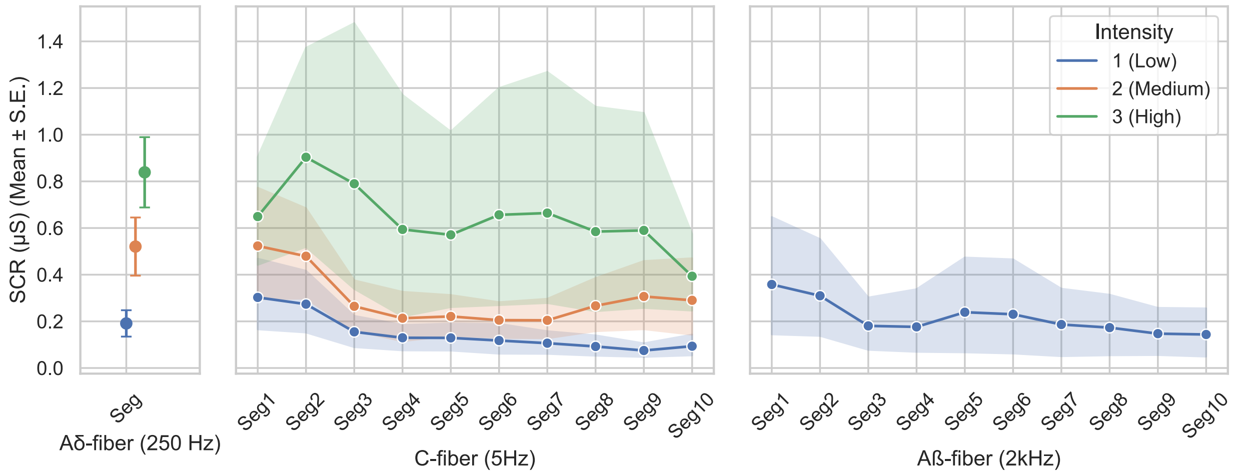

(b)

Supplementary Figure 2. Mean ± SEM of (a) nSCL and (b) SCR across time segments for each ECS intensity. Data points represent group means; error bars indicate between-subject variability.

2 Analysis on sensitive time-varying electrodermal activity index

2.1 TVSymp and MTVSymp Computation

In addition to skin condcuctance response (SCR) and skin conductance level (SCL), we also computed time-varying electrodermal-activity (EDA) indices, include TVSymp (Posada-Quintero et al., 2016) and modified TVSymp (MTVSymp) (Kong et al., 2021), both of which have demonstrated high sensitivity for assessing pain in experimental and clinical settings. The computation of TVSymp includes two steps: 1) variable frequency complex demodulation (Wang et al., 2006) to obtain and reconstruct EDA signals in the frequency range 0.08–0.24 Hz and 2) Hilbert transform to obtain instantaneous amplitudes of the reconstructed signals. A detailed description of this process is provided in (Posada-Quintero et al., 2016). The modified TVSymp (MTVSymp) was designed to detect rapid changes in sympathetic nervous system (SNS) activity to accentuate pain-associated EDA and minimize other responses using the following formula (Kong et al., 2021).

$$MTVSymp[x] = max(0, TVSymp[x] - \mu_{5sec})$$

where $\boldsymbol{\mu_{5sec}}$ represents for the average of immediately preceding 5-second of TVSymp values.

2.2 TVSymp and MTVSymp Results

A two-way repeated-measures ANOVA with factors Time and Intensity showed significant main effects of Intensity ($F(3,839) = 58.4, p < .001$), Time ($F(10,839) = 19.2, p < .001$), and their interaction ($F(29,839) = 1.6, p = .023$) for TVSymp. For MTVSymp, a two-way repeated-measures ANOVA with factors Time and Intensity showed significant main effects of Intensity ($F(3,839) = 22.2$, $p < .001$), Time ($F(10,839) = 19.1, p < .001$), and the Time × Intensity interaction ($F(29,839) = 2.2, p <.001$).

Supplementary Table 1 summarizes the statistical analyses of TVSymp and MTVSymp, and Supplementary Figure 3 illustrates their overall temporal trends. The first two segments (~15 seconds) showed comparable TVSymp amplitudes, followed by a significant decline for the remainder of the stimulation period at both Intensity 2 (p<.05) and Intensity 3 (p<.001). MTVSymp also showed a similar pattern, with a significant drop after the first segment (~10 s) for Intensities 2 and 3 (p<.05).

Both TVSymp and MTVSymp exhibited significantly higher values during the first segment compared with the Aβ control setting for Intensities 2 and 3 (p<.05). Notably, MTVSymp recovered at Segment 10, exhibiting significantly higher values than the Aβ-fiber control condition at Intensity 3. TVSymp showed a similar overall trend but with a more gradual transition. These results suggest that MTVSymp provides higher temporal precision in capturing rapid sympathetic fluctuations compared with TVSymp.

Supplementary Table 1. Statistical analysis on TVSymp and MTVSymp

	C-fiber Intensity	Segments with higher EDA than Aβ	Segments with lower EDA than Aδ
TVSymp	1		
	2	1,2 *	3-10 *
	3	1,2,8,9,10 *	3-10 **
MTVSymp	1		
	2	1 **	2-10 *
	3	1,10 *	2-10 *

**p*<.05, ***p*<.001

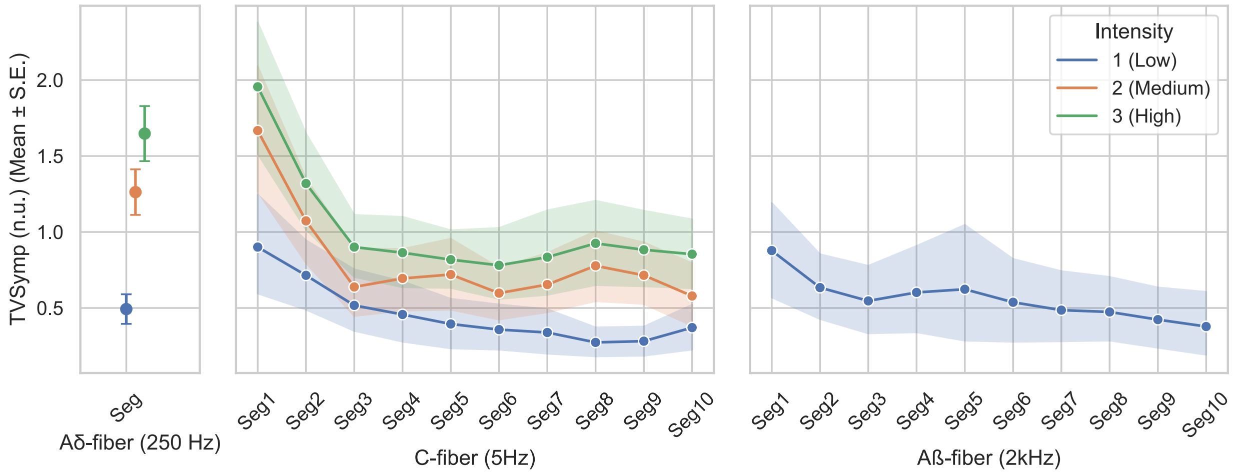

(a)

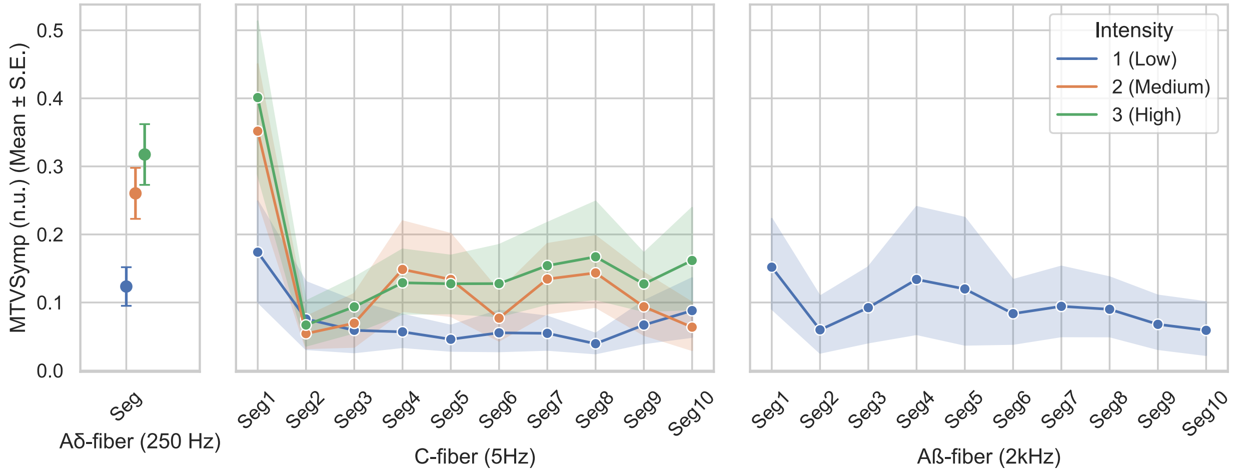

(b)

Supplementary Figure 3. Mean ± SEM of (a) TVSymp and (b) MTVSymp across time segments for each ECS intensity. Data points represent group means; error bars indicate between-subject variability.

Supplementary Figure 4 shows the results of the repeated-measures correlation analyses for TVSymp and MTVSymp. Stronger associations were observed under the Aδ-fiber setting compared with all timepoints in the C-fiber setting, with r = 0.69 ($p < .001$) for TVSymp and $r = 0.59$ ($p < .001$) for MTVSymp. For the C-fiber setting, TVSymp showed fair-to-moderate correlation coefficients during the first two segments (r = 0.42 and 0.54, p < .05) and the last four segments ($r = 0.36–0.49$, $p < .05$), whereas the middle segments exhibited poor, non-significant correlations ($r = 0.18–0.28$, p = n.s.). MTVSymp showed a fair correlation during the first segment ($r = 0.48$, $p < .05$) and fair correlations during Segments 6–8 ($r = 0.32–0.40$, $p<.05$). Overall, TVSymp and MTVSymp exhibited meaningful correlations during Aδ fiber stimulation and the initial phase of C-fiber stimulation, which may also involve partial Aδ fiber activation. However, their correlations across intensities became unstable during the middle phase of stimulation. This instability may be due to the inherent limitation of TVSymp, which extracts activity only within 0.08–0.24 Hz frequency band and thus may loss information relevant to C-fiber activation. Future work should explore new EDA-derived indices better suited for capturing sympathetic dynamics during tonic ECS stimulation.

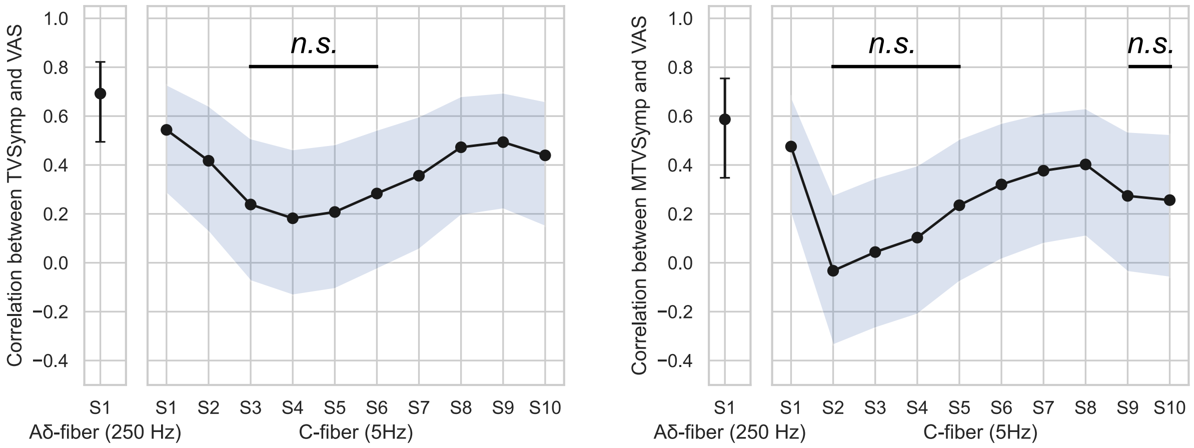

(a) (b)

Supplementary Figure 4. Repeated measures correlation between (a) TVSymp and VAS, and (b) MTVSymp and VAS at each time point. The error-bars and blue shades indicate 95% confidence intervals. All timepoints in panel (a) except those between S3 and S6, and all time points in panel (b) except those between S2 and S5 and between S9 and S10, showed significant correlation ($p<.05$).

While we focused on SCR and SCL, which are the most commonly used EDA features, other indices have also demonstrated high sensitivity in pain assessment, including the time-varying index of EDA (TVSymp) (Posada-Quintero et al., 2016) and its modified version (MTVSymp) (Kong et al., 2021). These features are derived using specific frequency bands that are highly sensitive to SNS activity, thereby accentuating SNS-associated activity compared with traditional SCR and SCL measures. Because our primary objective was to examine changes in SNS activation rather than to quantify pain itself, these results are presented in Supplemental Digital Content. To summarize, both TVSymp and MTVSymp showed similar performance for Aδ-fiber–associated stimulation (i.e., 250 Hz and the initial phase of 5 Hz stimulation). However, their correlations across intensities became unstable during the middle phase. This suggests that previously established EDA-based modeling approaches may be limited in characterizing long-term pain dynamics or C-fiber–dominated responses. Future algorithmic developments should account for these limitations to improve modeling accuracy, particularly for therapeutic and pharmaceutical applications in chronic pain research.

3 Validation of the custom smartphone application for continuous VAS assessment.

To validate the smartphone application developed for continuous VAS recording, we compared the average smartphone-derived VAS during long-term stimulation (60 s) with verbally reported VAS ratings obtained after each stimulation. Linear mixed-effects models were used, with stimulation intensity included as a covariate and participant modeled as a random intercept to account for repeated measures, as follows:

$$Smartphone\ VAS\ average \sim Verbal\ VAS + Intensity +\ (1 \mid Participant)$$

Smartphone-derived VAS showed a strong association with verbal VAS ratings ($F(1, 73.8) = 1644$, p <.001), after accounting for stimulation intensity and repeated measures within participants.

4 Linear mixed-effect model (LMER) diagnostics for the main models

Normal Q–Q plots indicated approximate normality for the VAS model, whereas EDA-based models (SCR and nSCL) exhibited modest right-tail deviations (Supplementary Fig. 5). Such deviations are consistent with the asymmetric and positively skewed characteristics of EDA and increased physiological variability at higher stimulus intensities. Residual-versus-fitted plots for VAS and SCR (Supplementary Fig. 5a–b) showed mild heteroscedasticity and a lower-bound constraint at small fitted values, reflecting the non-negative and bounded nature of these outcome measures. In contrast, nSCL was normalized at the first time point and therefore did not exhibit a lower-bound constraint in the residual-versus-fitted plot (Supplementary Fig. 5c). Overall, no major violations of linear mixed-effects model assumptions were observed.

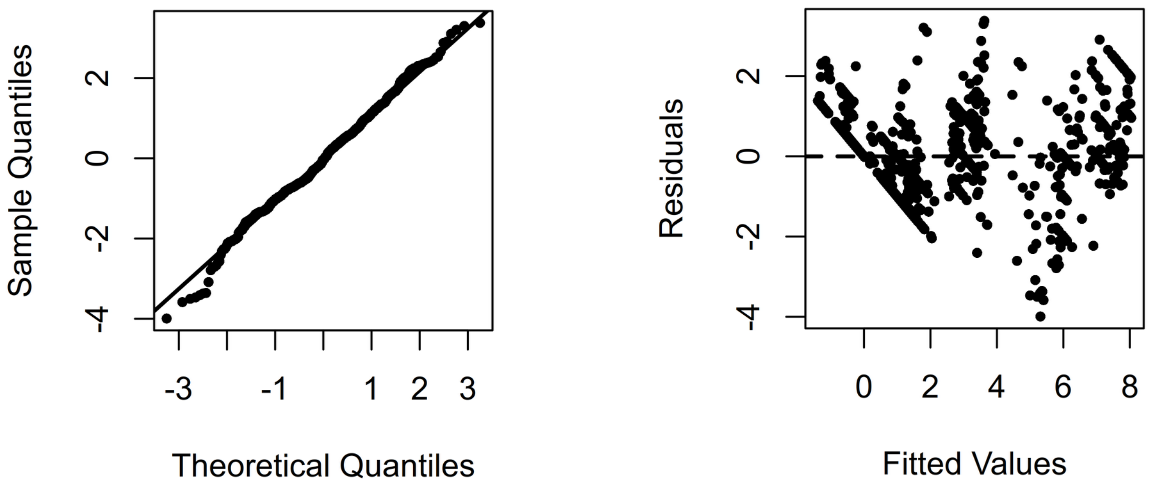

(a)

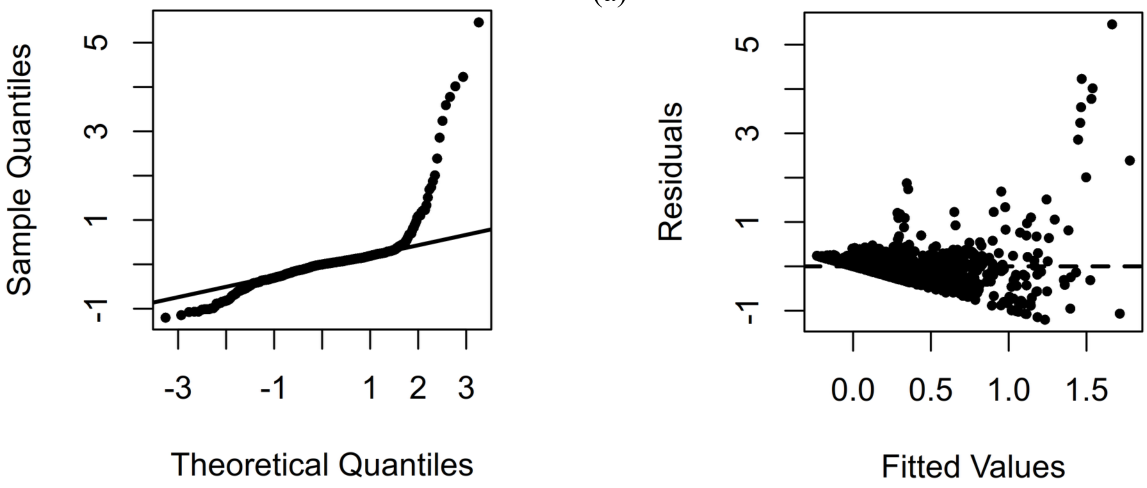

(b)

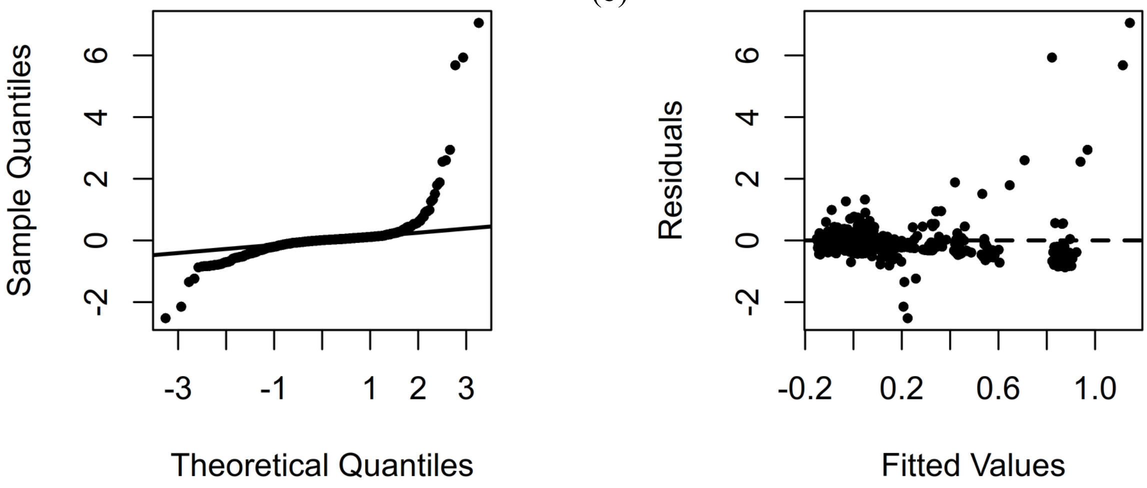

(c)

Supplementary Figure 5. Diagnostic plots (Q–Q plots, left; residuals versus fitted values, right) for the linear mixed-effects models predicting (a) VAS, (b) SCR, and (c) SCL from time, stimulus intensity, and their interaction, with participant included as a random intercept.

5. Effect of Biological Sex

To evaluate potential effects related to biological sex, sex was included as a fixed effect in additional linear mixed-effects models, as follows:

$$VAS, SCR, or\ SCL \sim Time\ \times\ Intensity \times Biological\ Sex + (1\ |Participant)$$

Biological sex did not show a significant main effect in pain scores and EDA indices; however, a modest sex-by-intensity interaction was consistently observed in pain scores ($F(3,797) = 5.9$, $p<.001$) SCR ($F(3,797) = 3.5$, $p<.05$), and nSCL ($F(3,797) = 5.6$, $p<.001$). Exploratory post-hoc analyses suggested that this interaction was most pronounced at the highest intensity for SCL (Supplementary Figure 6). No sex-dependent temporal effects were observed in the statistical models. However, these exploratory finding should be interpreted cautiously given the unbalanced and small sample size (14 male, 7 female).

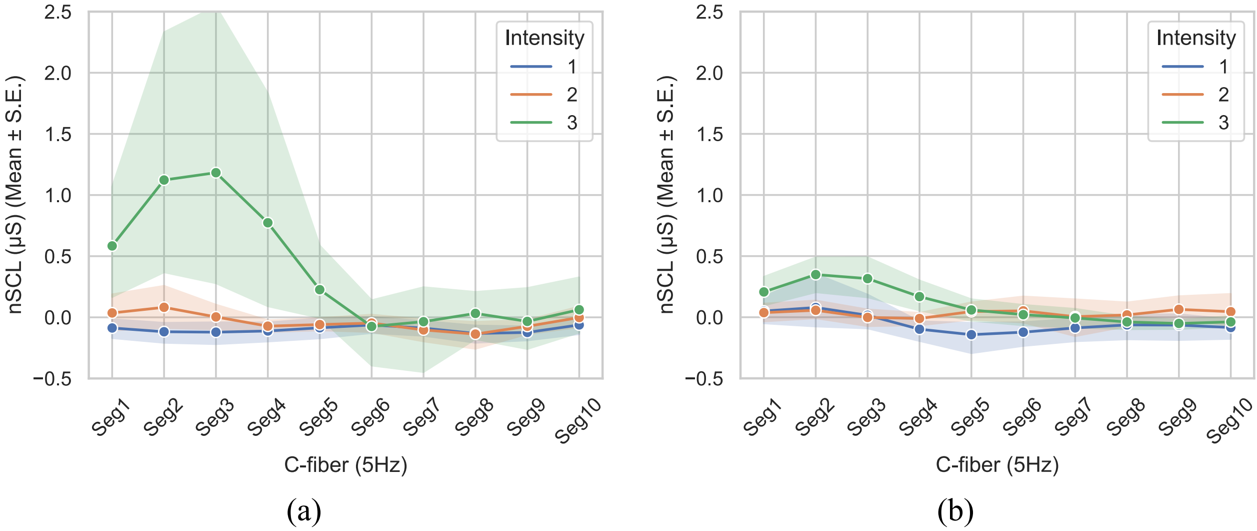

Supplementary Figure 6. Mean ± SEM of (a) males (N=14) and (b) females (N=7) across time segments for each ECS intensity. Data points represent group means; error bars indicate between-subject variability.

References

Kong, Y., Posada-Quintero, H. F., and Chon, K. H. (2021). Sensitive physiological indices of pain based on differential characteristics of electrodermal activity. *Trans. Biomed. Eng.* 68, 3122–3130.

Posada-Quintero, H. F., Florian, J. P., Orjuela-Cañón, Á. D., and Chon, K. H. (2016). Highly sensitive index of sympathetic activity based on time-frequency spectral analysis of electrodermal activity. *American Journal of Physiology-Regulatory, Integrative and Comparative Physiology* 311, R582–R591. doi: 10.1152/ajpregu.00180.2016

Wang, H., Siu, K., Ju, K., and Chon, and K. H. (2006). A High Resolution Approach to Estimating Time-Frequency Spectra and Their Amplitudes,. *Ann Biomed Eng* 34, 326–338.
